# Aging-Dependent Altered Transcriptional Programs Underlie Activity Impairments in Human C9orf72-Mutant Motor Neurons

**DOI:** 10.3389/fnmol.2022.894230

**Published:** 2022-06-14

**Authors:** Daniel Sommer, Sandeep Rajkumar, Mira Seidel, Amr Aly, Albert Ludolph, Ritchie Ho, Tobias M. Boeckers, Alberto Catanese

**Affiliations:** ^1^Institute of Anatomy and Cell Biology, Ulm University School of Medicine, Ulm, Germany; ^2^Department of Neurology, Ulm University School of Medicine, Ulm, Germany; ^3^German Center for Neurodegenerative Diseases (DZNE), Ulm, Germany; ^4^Center for Neural Science and Medicine, Cedars-Sinai Medical Center, Los Angeles, CA, United States; ^5^Board of Governors Regenerative Medicine Institute, Cedars-Sinai Medical Center, Los Angeles, CA, United States; ^6^Department of Biomedical Sciences, Cedars-Sinai Medical Center, Los Angeles, CA, United States; ^7^Department of Neurology, Cedars-Sinai Medical Center, Los Angeles, CA, United States

**Keywords:** hiPSC, ALS (amyotrophic lateral sclerosis), motor neuron (MN), synapse, transcriptomic (RNA-Seq), neuronal excitability

## Abstract

Amyotrophic Lateral Sclerosis (ALS) is an incurable neurodegenerative disease characterized by dysfunction and loss of upper and lower motor neurons (MN). Despite several studies identifying drastic alterations affecting synaptic composition and functionality in different experimental models, the specific contribution of impaired activity to the neurodegenerative processes observed in ALS-related MN remains controversial. In particular, contrasting lines of evidence have shown both hyper- as well as hypoexcitability as driving pathomechanisms characterizing this specific neuronal population. In this study, we combined high definition multielectrode array (HD-MEA) techniques with transcriptomic analysis to longitudinally monitor and untangle the activity-dependent alterations arising in human C9orf72-mutant MN. We found a time-dependent reduction of neuronal activity in ALS^C9orf72^ cultures occurring as synaptic contacts undergo maturation and matched by a significant loss of mutant MN upon aging. Notably, ALS-related neurons displayed reduced network synchronicity most pronounced at later stages of culture, suggesting synaptic imbalance. In concordance with the HD-MEA data, transcriptomic analysis revealed an early up-regulation of synaptic terms in ALS^C9orf72^ MN, whose expression was decreased in aged cultures. In addition, treatment of older mutant cells with Apamin, a K^+^ channel blocker previously shown to be neuroprotective in ALS, rescued the time-dependent loss of firing properties observed in ALS^C9orf72^ MN as well as the expression of maturity-related synaptic genes. All in all, this study broadens the understanding of how impaired synaptic activity contributes to MN degeneration in ALS by correlating electrophysiological alterations to aging-dependent transcriptional programs.

## Introduction

Amyotrophic lateral sclerosis (ALS) is a fatal neurodegenerative disease mainly affecting the spinal motor neurons (MN) that leads to progressive muscular denervation and eventually death by respiratory failure (Hardiman et al., 2017). Despite the recent advances in our understanding of the genetic causes of this disease, the exact molecular pathomechanisms leading to the selective loss of MN are not fully understood. In fact, the observations showing that ALS-related genes such as *C9orf72* (the most frequent genetic cause of ALS; DeJesus-Hernandez et al., 2011), *TARDBP, SOD1* and *FUS* are involved in several and different biological processes (e.g., RNA metabolism, autophagy, DNA repair; Gao et al., 2017; Hardiman et al., 2017) makes the pathological landscape of this disorder extremely puzzling. As a result, patients are still missing a reliable and effective therapeutic strategy as ALS remains incurable.

The establishment of human induced pluripotent stem cells (hiPSC) as an experimental platform for disease modeling and drug testing opened the possibility of performing large scale *in vitro* studies including a high number of patient-derived iPSC lines (Fujimori et al., 2018; Mertens et al., 2021). Nevertheless, contradictory results have also arisen from studies based on iPSC-derived neuronal models of ALS, in particular when focusing on activity, synaptic and electrophysiological alterations occurring in mutant MN. In line with the excitotoxicity model (Rothstein et al., 1990), it has been shown that dendritic accumulation of NMDA receptors, as well as increased expression of the AMPA subunit GluA1, confer higher vulnerability to glutamate and excitotoxicity in human C9orf72-mutant MN (Selvaraj et al., 2018; Shi et al., 2018). Moreover, another study showed that reduced delayed-rectifier K^+^ current in SOD1-mutant human MN leads to hyperactivity and treatment with the K^+^ channel opener Retigabine could reduce the aberrant firing and rescue neuronal survival (Wainger et al., 2014). In contrast, reduced neuronal activity has been observed in ALS-related human MN by various other groups: Naujock and colleagues reported less spontaneous firing and synaptic inputs in human MN with *FUS* and *SOD1* mutations than in cultures from healthy controls (Naujock et al., 2016). In addition, another study linked reduced excitability in C9orf72-mutant MN to increased expression of K^+^ channels (Sareen et al., 2013) and, more recently, the K^+^ channel blocker Apamin proved neuroprotective in human C9orf72-mutant MN (and Drosophila) by two independent research teams (Castelli et al., 2021; Catanese et al., 2021).

Interestingly, these discrepancies seem to arise from the different stages of cell culture at which the experiments have been performed. Indeed, the studies suggesting hyperactivity as a major driving pathomechanism in ALS have been mainly performed by analyzing MN within the first 4 weeks of culture, whereas reduced firing properties are observed at later stages of *in vitro* maturation. Notably, this dynamic time-dependent switch from hyper- to hypoactivity in MN cultures from ALS patients has been well described by Devlin and colleagues, who monitored the electrophysiological changes occurring over time in C9orf72- and TARDBP-mutant MN and described a progressive loss of excitability in mutant cultures (Devlin et al., 2015). This indicates that the maturation stage of human MN is a crucial factor to be considered for the identification of activity phenotypes that might resemble *in vitro* what is observed in patients. Indeed, despite hiPSC-derived MN resembling a maturation status closer to the embryonic rather than the adult stage, aging contributes even *in vitro* to pathology progression (Higelin et al., 2016; Ho et al., 2016). Thus, a deeper understanding of the biological and molecular changes occurring in iPSC-derived MN upon maturation might contribute to elucidate which pathological alterations are indeed of translational relevance and targetable for the development of novel therapies.

Since synapses are a crucial structure involved in neuronal firing, we investigated in this study to which extent the synaptic transcriptome might represent a reliable readout to pinpoint a specific and relevant maturation state of hiPSC-derived MN for the modeling of activity-related alterations in ALS.

## Materials and Methods

### Human Induced Pluripotent Stem Cells

The cell lines used in this study have already been described in Catanese et al. (2021): from the two healthy controls, one was a 45-years old female and the other one a 49-years old male individual (Cedars-Sinai CS0YX7iCTR-nxx). Both mutant lines were purchased from the Induced Pluripotent Stem Cell Core of Cedars-Sinai (Los Angeles, CA, United States) and were obtained from a 46-years old male (ALS^C9orf72^ II; CS29iALS-C9nxx) and a 51-years old female patient (ALS^*C*9orf72^ III; CS30iALS-C9nxx). HiPSCs were cultured in mTeSR1 medium (Stem Cell Technologies, Vancouver, BC, Canada; 85850) supplemented with 1% antibiotic-antimycotic (Thermo Fisher Scientific, Waltham, MA, United States; 15240062) at 37°C (5% CO2, 5% O2) on Matrigel^®^ -coated (Corning, Corning, NY, United States; 354277) 6-well plates (Corning, Corning, NY, United States; 353046). The medium was exchanged daily after manually removing the spontaneously differentiated cells. Once the colonies had reached 80% confluence, Dispase (Stem Cell Technologies, Vancouver, BC, Canada; 07913) was used to detach them and cells were passaged in 1:3 or 1:6 split ratio. Karyotyping was performed (after treatment of hiPSCs for 2 h with 1.5 M colchicine, Eurobio Scientific, Les Ulis, France; CCHCLC00-JA) to exclude chromosomal aberrations. The presence of contaminants was monitored with DAPI staining and the Cell Culture Contamination Kit from Molecular Probes (Thermo Fisher Scientific, Waltham, MA, United States; C-7028).

### Motor Neuron Differentiation and Culture

Motor neurons were differentiated from hiPSCs as previously described (Catanese et al., 2021). Briefly, hiPSC colonies were detached with Dispase (Stem Cell Technologies, Vancouver, BC, Canada; 07913) and transferred to suspension culture in ultra-low attachment flasks (Corning, Corning, NY, United States; 4616) for 3 days in order to allow the formation of embryoid bodies (EBs) in neuronal induction medium (DMEM/F12 (Thermo Fisher Scientific, Waltham, MA, United States; 31331028) + 20% knockout serum replacement (Thermo Fisher Scientific, Waltham, MA, United States; 10828028) + 1% NEAA (Thermo Fisher Scientific, Waltham, MA, United States; 11140035) + 1% β-mercaptoethanol (Merck Millipore, Burlington, MA, United States; ES-007-E) + 1% antibiotic-antimycotic (Thermo Fisher Scientific, Waltham, MA, United States; 15240062) + SB-431542 10 μM (Stem Cell Technologies, Vancouver, BC, Canada; 72232) + Dorsomorphin 1 μM (Tocris Bioscience, Bristol, United Kingdom; 3093) + CHIR 99021 3 μM (Stem Cell Technologies, Vancouver, BC, Canada; 72052) + Purmorphamine 1 μM (Miltenyi Biotec, Bergisch Gladbach, Germany; 130-104-465) + Ascorbic Acid 200 ng/μl (Sigma-Aldrich, St. Louis, MO, United States; A4403) + cAMP 10 μM (Sigma-Aldrich, St. Louis, MO, United States; D0260) + 1% NeuroCult without vitamin A (Stem Cell Technologies, Vancouver, BC, Canada; 05731) + 0.5% N2 (Thermo Fisher Scientific, Waltham, MA, United States; 17502048)). On the fourth day, medium was changed to MN medium (DMEM/F12 + 24 nM sodium selenite (Sigma-Aldrich, St. Louis, MO, United States; S5261) + 16 nM progesterone (Sigma-Aldrich, St. Louis, MO, United States; P8783) + 0.08 mg/ml apotransferrin (Sigma-Aldrich, St. Louis, MO, United States; T2036) + 0.02 mg/ml insulin (Sigma-Aldrich, St. Louis, MO, United States; 91077C) + 7.72 μg/ml putrescine (Sigma-Aldrich, St. Louis, MO, United States; P7505) + 1% NEAA + 1% antibiotic-antimycotic + 50 mg/ml heparin (Sigma-Aldrich, St. Louis, MO, United States; H4784) + 10 μg/ml of the neurotrophic factors BDNF, GDNF, and IGF-1 (PreproTech, Cranbury, NJ, United States; 450-02, 450-10 and 100-11) + SB-431542 10 μM + Dorsomorphin 1 μM + CHIR 99021 3 μM + Purmorphamine 1 μM + Ascorbic Acid 200 ng/μl + Retinoic Acid 1 μM (Tocris Bioscience, Bristol, United Kingdom; 0695) + cAMP 1 μM + 1% NeuroCult without vitamin A + 0.5% N2). On the eighth day in suspension culture, EBs were dissociated into single cells with Accutase (Sigma-Aldrich, St. Louis, MO, United States; A6964) and plated onto 6-well plates (Corning, Corning, NY, United States; 353046), 24-well μ-Plates (ibidi, Gräfelfing, Germany; 82426) and MaxOne chips (see below) pre-coated with Growth Factor Reduced Matrigel^®^ (Corning, Corning, NY, United States; 356231; diluted 1:100 according to the manufacturer’s instructions). Cells were plated in MN maintenance medium (DMEM/F12 containing 1% antibiotic-antimycotic solution + 1% NEAA + 24 nM sodium selenite + 16 nM progesterone + 0.08 mg/ml apotransferrin + 0.02 mg/ml insulin + 7.72 μg/ml putrescine + 50 mg/ml heparin + 2% NeuroCult without vitamin A + 200 ng/μl AA + 10 μM each of BDNF, GDNF and IGF-1 + 1 μM purmorphamine + 1 μM cAMP + 1 μM RA) and except for MaxOne chips (see below) a 50% medium change was performed twice per week without the addition of any antimitotic agent. The potassium channel blocker Apamin (Tocris Bioscience, Bristol, United Kingdom; 1652) was administered in a final concentration of 500 nM for three consecutive days. In MEA experiments, cultures were first recorded, then treated with Apamin, and again recorded after K^+^ channel blockade.

Cultures contained 50% of MAP2 neurons (out of which 90% being Chat-positive cells) and have been previously characterized for cell composition in Catanese et al. (2021).

### Multielectrode Array

Prior to plating, MaxWell MaxOne chips (MaxWell Biosystems AG, Zurich, Switzerland) were treated with sterile filtered Tergazyme^®^ (Alconox, New York City, NY, United States; 1304-1) 1% solution at 37°C overnight in order to increase the hydrophilicity of the surface. After removal of Tergazyme^®^ solution and disinfection with 70% ethanol for 15–20 min, the chips were rinsed three times with PBS (-/-; Thermo Fisher Scientific, Waltham, MA, United States; 14190094) before coating each with 500 μl of Growth Factor Reduced Matrigel^®^ (Corning, Corning, NY, United States; 356231; diluted 1:100) for 2 h at 37°C. Afterward the coating solution was removed and 90,000 cells per chip were seeded in 500 μl MN maintenance medium. One day after plating 500 μl of MN maintenance medium were added and starting from DIV5 a 25% medium change was conducted three times per week. Presented longitudinal data originate from a minimum of three independent differentiations per cell line. Electrophysiological parameters were obtained using a MaxOne HD-CMOS MEA system (MaxWell Biosystems AG, Zurich, Switzerland). The system’s gain was set to 512× with a high-pass filter from 300 Hz and a spike threshold of 5.00. During recordings the system was kept inside a cell culture incubator at 37°C and 5% CO2. Starting from DIV14, the activity of the chips was monitored with weekly full-sensor “Activity Scan” assays in MaxLab Live software (data not shown) with a record time of 20 s per electrode. If chips presented less than 2.5% active electrodes in two subsequent assays, chips were discarded and not considered for further analysis. Additionally, we performed “Network Scan” assays (in MaxLab Live software) on DIV21 and DIV42, if the chip showed more than 2.5% active electrodes in the preceding full-sensor “Activity Scan” assay at the respective time point. Data shown in this paper was exclusively obtained from the “Network Scan” assays, where only the most active subset of electrodes per chip (as evaluated by a built-in algorithm in MaxLab Live software, based on the firing rate) was recorded over a period of 300 s. In order to allow a paired comparison within genotypes for different timepoints, incomplete datasets (chips showing less than 2.5% active electrodes for either DIV21 or DIV42) were not considered in the final analysis. To evaluate the effect of Apamin, one “Network Scan” assay was carried out as a baseline and a second one with the same settings and electrode configuration was conducted after drug administration.

### qRT-PCR

RNA was isolated from MNs using the RNeasy Mini kit (Qiagen, Hilden, Germany; 74106) according to the manufacturer’s instructions. At first, strand synthesis and quantitative real-time-PCR amplification were performed in a one-step, single-tube format using the Rotor-Gene^®^ SYBR Green RT-PCR kit (Qiagen, Hilden, Germany; 204174) as described by the manufacturer in a total volume of 20 μl. The following settings were used: 10 min at 55°C and 5 min at 95°C, followed by 40 cycles of PCR for 5 s at 95°C for denaturation and 10 s at 60°C for annealing and elongation (one-step). The SYBR Green I reporter dye signal was measured against the internal passive reference dye (ROX) to normalize non-PCR-related fluctuations. Resulting expression data were normalized to the levels of HMBS, used as a housekeeping gene. The Rotor-Gene Q software (version 2.0.2) was used to calculate the cycle threshold values. All experiments were performed in 3 technical replicates. All Primers used in this study are commercially available (Qiagen QuantiTect Primer Assays, Qiagen, Hilden, Germany; 249900; validated primers without sequence information): ATP6V0A4 (QT00016828), BSN (QT00028819), GRIA3 (QT00050092), GRIN2A (QT00050379), SNAP91 (QT00055139), SYNGR1 (QT00041881), SYT2 (QT00069293), SYT4 (QT00003269), SYT3 (QT00079989).

### Total mRNA Sequencing

Samples were collected from each cell line at DIV21 and 42 from 4 independent cultures (obtained from two independent differentiations) and pooled after isolation to reduce batch variations. RNA was isolated using the RNAeasy Mini kit (Qiagen, Hilden, Germany; 74106) following the instructions provided by the manifacturer and the concentration was estimated with a NanoDrop 2000 spectrophotometer (Thermo Fisher Scientific, Waltham, MA, United States). Whole-transcriptome analysis was performed at the Cambridge Sequencing Center (United Kingdom) of Novogene. RNA quality was assessed with an Agilent 2100 bioanalyzer system (Agilent, Santa Clara, CA, United States) and a total amount of 1 μg of mRNA per sample, isolated from total RNA by using poly-T oligo-attached beads, was used as input material for the RNA sample preparations. Sequencing libraries were generated using NEBNext^®^ UltraTM RNA Library Prep Kit for Illumina^®^ (NEB, Ipswich, MA, United States) following manufacturer’s recommendations. Fragmentation was carried out using divalent cations under elevated temperature in NEBNext First Strand Synthesis Reaction Buffer (5X). After fragmentation, the first strand cDNA was synthesized using random hexamer primers followed by the second strand cDNA synthesis using either dUTP for directional library or dTTP for non-directional library. The libraries were sequenced on an Illumina^®^ platform (Illumina, San Diego, CA, United States) at a sequencing depth of 9 Gb using 150 base paired-end reads, which were mapped referring to the primary assembly of the human genome (hg38) available in Ensembl. Alignment was performed using Hisat2 v2.0.5 and gene expression levels were quantified using featureCounts v1.5.0-p3 to count the reads number mapped for each gene. Afterward, Fragments Per Kilobase of transcript per Million mapped reads (FPKM) for each gene were calculated considering the gene length and the corresponding reads count mapped.

### LDH Assay

In order to quantify cell stress and death within the cultures, CyQUANT™ LDH Cytotoxicity Assay (Thermo Fisher Scientific, Waltham, MA, United States; C20300) was carried out according to manufacturer’s instruction, quantifying the leaked LDH (released from damaged cells) in the culture medium. Briefly, 50 μl of medium were collected from each culture well and mixed with 50 μl of Reaction Mixture provided with the kit in a 96-well plate at room temperature protected from light. After 30 min of incubation, 50 μl of Stop Solution (provided with the kit) were added to each well. Subsequently, the absorbance of the Formazan-dye produced by the reaction was measured at 490 nm in a Gen5 microplate reader (BioTek Instruments, Winooski, VT, United States). Additionally, the absorbance at 680 nm was quantified and subtracted to detect and control background signals.

### Immunocytochemistry

Immunocytochemistry was performed as previously described (Catanese et al., 2019). MN cultured in microscopable multiwell-plates were fixed for 7 min with 4% paraformaldehyde (Sigma-Aldrich, St. Louis, MO, United States; P6148) solution containing 10% sucrose (Carl Roth, Karlsruhe, Germany; 4621.1). After a 2 h incubation in blocking solution (PBS (-/-; Thermo Fisher Scientific, Waltham, MA, United States; 14190094) + 10% Goat Serum (Merck Millipore, Burlington, MA, United States; S26-100ML) + 0.2% Triton X-100 (Sigma-Aldrich, St. Louis, MO, United States; T8787)), primary antibodies were diluted in an equally composed solution and cells were incubated overnight at 4°C. In the next step, cells were washed three times for 30 min with PBS (-/-; Thermo Fisher Scientific, Waltham, MA, United States; 14190094) before incubation with secondary antibodies diluted 1:1000 in PBS (-/-; Thermo Fisher Scientific, Waltham, MA, United States; 14190094) for 2 h at room temperature. Following three further washing steps with PBS (-/-; Thermo Fisher Scientific, Waltham, MA, United States; 14190094), cells were mounted using ProLong™ Gold Antifade Mountant with DAPI (Thermo Fisher Scientific, Waltham, MA, United States; P36935) mixed with ibidi Mounting Medium (ibidi, Gräfelfing, Germany; 50001). The following primary antibodies have been used: MAP2 (diluted 1:2000; EnCor Biotechnology, Gainesville, FL, United States; CPCA-MAP2), Homer1 (diluted 1:500; Abcam, Cambridge, United Kingdom; ab184955), Bassoon (diluted 1:500; ENZO Life Sciences, Farmingdale, NY, United States; ADI-VAM-PS003-D). The following secondary antibodies from Thermo Fisher Scientific (Waltham, MA, United States) were used: goat anti-Mouse Alexa Fluor 488 (A-11001), goat anti-Rabbit Alexa Fluor 568 (A-11011) and goat anti-Chicken Alexa Fluor 647 (A32933).

### Microscopy and Image Analysis

Confocal microscopy was performed with a laser-scanning microscope (Leica DMi8; Leica, Wetzlar, Germany) equipped with an ACS APO 63 × oil DIC immersion objective. Images were obtained using the LasX software (Leica, Wetzlar, Germany), with a resolution of 1,024 × 1,024 pixels and a number of Z-planes (step size of 0.3) enough to span the complete neuron. The mean intensity of Bassoon and Homer1 spots was calculated by drawing a 20 μm-long region of interest along 3 different dendrites belonging to each neuron considered. To identify synaptic contacts, the colocalization between pre- and postsynaptic markers (Bassoon and Homer1, respectively) was analyzed using Imaris software (Bitplane, Zurich, Switzerland; version 9.7.2). First, a surface of reference was drawn in the MAP2 channel with the Surface tool. Afterward, the puncta for each marker were detected semi-automatically in the respective channel (with the Spots tool), and the interaction between the two proteins was accepted within a minimum distance of 0.8 μm between the center of the respective spots and with a maximum distance of 1 μm from the dendrite. The computational parameters and post-acquisition modifications were equally applied to analyzed pictures belonging to the same experiments and for figure display.

### Data and Statistical Analysis

To compare two independent groups (genotypes or treatment), we used unpaired *t*-test with Welch correction in case of normally distributed data and non-parametric Mann–Whitney test in case of non-normal distribution.

The analysis of synaptic genes during spinal MN maturation was performed as previously described in Ho et al. (2016). Briefly, synaptic genes were subset from the composite data set (including transcriptomic profiles from samples ranging from human pluripotent cells, iPSC-motor neurons, fetal and adult spinal cords, and laser capture micro-dissected motor neurons from adult spinal cords) from Ho et al. (2016), log 2 transformed, and projected using principal component analysis. Using the program Cluster 3.0, these genes were clustered with centered average linkage, and the dendrogram and heatmap are presented using Java TreeView and Microsoft Excel, respectively. Protein-protein interaction was analyzed with STRING (version 11.5) and Cytoscape (version 3.9).

In the RNAseq experiments, the differential expression analysis between two conditions/groups (three replicates per condition and cell line) was performed using the DESeq2 R package and considering only transcripts with [FPKM] ≥ 1. The resulting *p*-values were adjusted using the Benjamini and Hochberg’s approach for controlling the False Discovery Rate (FDR). Genes with an adjusted *p*-value < 0.05 were assigned as differentially expressed. Gene Ontology (GO) enrichment analysis of differentially expressed genes was performed with g:Profiler (Raudvere et al., 2019). GO terms with corrected *p*-value less than 0.05 were considered as significantly enriched. Gene Set Enrichment Analysis (GSEA) was performed to computationally determine if a predefined gene set associated with a specific function might be significantly associated to a specific sample/biological state (Subramanian et al., 2005). Synaptic genes were filtered from the DEG lists using the SynGO analytical tool (Koopmans et al., 2019).

MEA recordings were analyzed with the analysis tools of the MaxLab Live software (MaxWell Biosystems AG, Zurich, Switzerland; version 21.1.26). We evaluated single electrode-parameters by activity analysis and synchronicity parameters by network analysis, using the data generated with “Network Scan” assay. Used settings are indicated in Table 1.

**TABLE 1:** Summary of the parameters used for MEA analysis.

Activity analysis	Firing Rate Threshold (Hz): 0.10; Amplitude Threshold (μV): 20.00; ISI Threshold (ms): 200
Network analysis	Smoothing window size (s): 0.30; Burst Detection Threshold: 1.20; Use Fixed Burst Detection Threshold: FALSE; Minimum Peak Distance (s): 1.00; Start-Stop Threshold: 0.30

To analyze the effect of Apamin on the activity properties of C9orf72-mutant cultures, the recordings were again analyzed using built-in tools of MaxLab Live software. Activity analysis was performed with the same settings as stated above. To improve comparability, the fixed burst detection threshold-option was used for the network analysis. The applied burst detection threshold for each chip was determined automatically in the pre-treatment condition and then manually set to the same value for the post-treatment condition. To counteract the effects of increased noise caused by generally higher activity in the post-treatment condition, the smoothing window size was raised to 0.5 s for both of the pre- and post-treatment network analysis. Settings apart from “Use fixed burst detection threshold” and “Smoothing window size” were left unchanged from what is stated in Table 1. Data were collected and statistically analyzed using Microsoft Excel (Microsoft, Redmont, WA, United States) and GraphPad Prism (GraphPad Software, San Diego, CA, United States; version 9.3.1). Values not matching the above-mentioned criteria (active electrodes > 2.5%, complete dataset for both time points) were excluded. Because of the data being not normally distributed, these were statistically analyzed with non-parametric tests. Therefore, four non-parametric *t*-tests covering all relevant comparisons (within time points/between genotypes and between time points/within genotypes) were run for each parameter, followed by adjusting the *p*-values for multiple testing by Bonferroni’s correction (Supplementary Table 1).

## Results

### Synaptic Transcriptome Defines the Maturation Status of Human Motor Neurons

**FIGURE 1:**
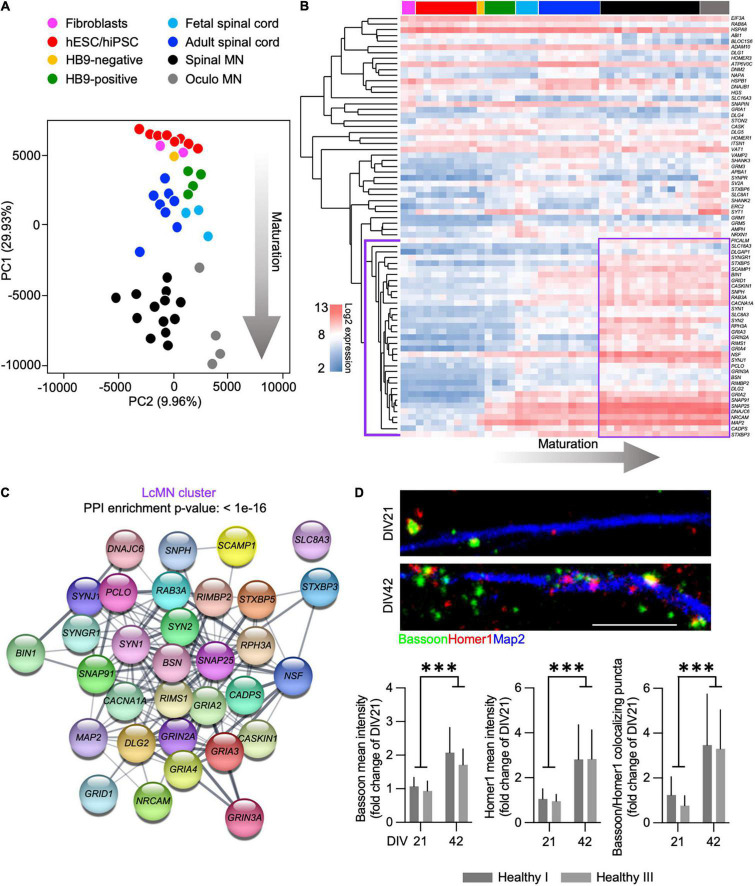
Expression of synaptic transcripts shapes differentiation and maturation of human motor neurons. (A) PCA based on the synaptic transcriptome showing the dynamics of maturation (arrow) from undifferentiated stem cells to laser-captured adult MN along PC1. (B) Unsupervised hierarchical clustering of 74 synaptic genes highlighting the LcMN cluster of transcripts (outlined in violet) whose higher expression defines adult motor neurons. (C) Protein-protein interaction analysis performed with STRING identifies a significant (*p* < 10^–16^) functional networking between the genes of the LcMN cluster. (D) Confocal images and analysis of the synaptic markers Bassoon, Homer1 and their colocalization in DIV21 and DIV42 MN from healthy individuals showing a significant increase in the density of synaptic contacts upon maturation. Scale bar: 5 μm. ^***^p < 0.001.

We analyzed the expression of synaptic genes by incorporating microarray data obtained from human fibroblasts, hiPSCs and hESCs, ESC-derived MN (expressing a MN-specific GFP:HB9 reporter and that were sorted in GFP-positive and GFP-negative samples), fetal and adult spinal cord, as well as laser-captured motor and oculomotor neurons (henceforth referred to as LcMN) (GSE75701; Ho et al., 2016). Principal component analysis (PCA) highlighted that PC1 could resolve the distribution of the different samples according to their maturation status (Figure 1A) by displaying their progression from embryonic to adult. Indeed, human iPSC and ESC clustered close to fibroblasts and HB9-negative sorted fractions from ESC-derived MN cultures. In addition, the synaptic transcriptome of cultured human MN was similar to those obtained from fetal and adult spinal cord samples. Although it has been previously demonstrated that iPSC-derived neuronal cultures resemble the properties of embryonic tissues rather than adult ones (Ho et al., 2016), these results can be explained by the low abundance of MN present in the spinal cord. Indeed, the LcMN clustered separately from all the other samples included in the analysis, thus representing the most mature stage of neuronal maturation according to synaptic transcripts.

To gain deeper information on the synaptic transcriptome defining the mature status of human MN, we ranked 74 synaptic genes according to their loading scores across the PC1 (transcripts with the most negative gene loading are the most highly expressed in the adult LcMN; Supplementary Table 2). Hierarchical clustering of these 74 genes (Figure 1B) revealed a group of 35 synaptic transcripts (LcMN cluster - outlined in violet) whose expression progressively increased upon maturation reaching the highest values in LcMN and, as expected, showed a strongly significant functional interaction (Figure 1C).

Since ALS is a late-onset neurodegenerative disease (Broussalis et al., 2018), cultured MN from ALS patients show altered transcriptional programs associated with aging (Ho et al., 2016) and the synaptic transcriptome of stem cell-derived MN does not resemble the one of adult LcMN, we asked which stage of differentiation might represent a valid choice for modeling *in vitro* the synaptic alterations observed in motor neuron disease. To this end, we evaluated the levels of the presynaptic marker Bassoon (which was identified within the LcMN cluster) and the postsynaptic scaffold Homer1 (whose expression also increased from ESC-derived MN to LcMN) in hiPSC-derived MN obtained from 2 healthy individuals after 3 (DIV21) and 6 weeks (DIV42) in culture. We observed a significant increase in the intensity of dendritic Homer1 and Bassoon upon aging and, accordingly, also the number of synapses (identified as colocalizing Bassoon:Homer1 spots) was higher in DIV42 MN than at DIV21 (Figure 1D). This indicated that synaptic composition and density might define relevant maturation stages for the investigation of ALS-related phenotypes in hiPSC-derived MN.

### ALS^C9orf72^ Cultures Display Age-Dependent Altered Electrophysiological Properties

**FIGURE 2:**
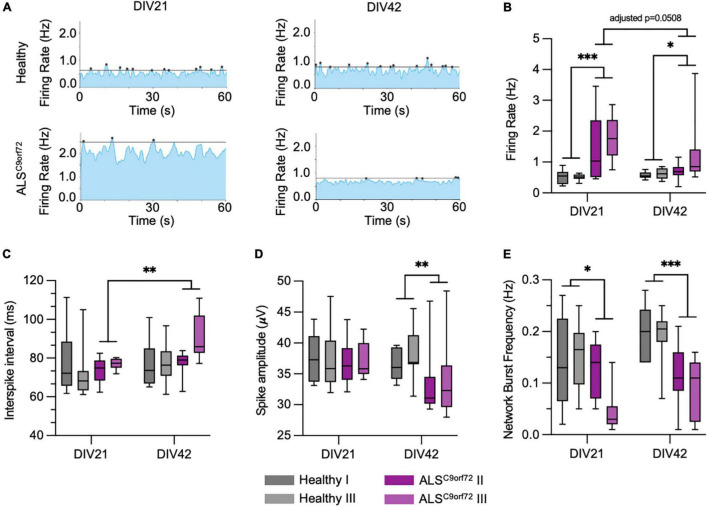
ALS^C9orf72^ motor neurons display age-dependent alterations in activity and network properties. (A) Representative network activity plots of Healthy and ALS^C9orf72^ MN at DIV21 and DIV42 (lines indicate the network burst detection threshold). (B) ALS^C9orf72^ MN display an early hyperactivity compared to controls at DIV21. At the later time point, the firing rate of both ALS^C9orf72^ lines drops to values closer to Healthy ones, even though the two ALS^C9orf72^ show a close-to-significance difference in this firing property (firing rate at DIV42, ALS^C9orf72^ II vs. ALS^*C*9*orf*72^ III: *p* = 0.074). (C) Within the ALS^C9orf72^ genotype, the interspike interval (ISI) during repetitive firing sequences significantly increases between the two time points. (D) ALS^C9orf72^ MN show a reduced spike amplitude at DIV42. (E) The network burst frequency is significantly lower in ALS^C9orf72^ than in Healthy MN at both time points. *p < 0.05; ^**^p < 0.01; ^***^p < 0.001.

To clarify the conflicting evidence on time dependent electrophysiological alterations in hiPSC-derived ALS^C9orf72^ MN, we longitudinally monitored cellular activity with a high definition multielectrode array (HD-MEA) system. We employed two previously published C9orf72-mutant hiPSC lines (henceforth ALS^C9orf72^; Catanese et al., 2021), carrying hexanucleotide repeat expansions of 6–8 and 2.7 kb length, respectively, and compared their firing properties to those of two gender- and age-matched healthy controls. The same cultures were measured at DIV21 and DIV42 (Figure 2A) representing, as suggested by the results presented above, an early and a late stage of synaptic maturation *in vitro*. At DIV21, we found a significantly higher firing rate in ALS^C9orf72^ MN compared to Healthy controls, which had dropped at DIV42. After the additional 3 weeks of maturation, the firing rate of ALS^C9orf72^ was indeed lower (*p* = 0.0508) than the one of the same cultures at DIV21, while it remained unchanged in control neurons (Figure 2B). Of note, the firing rate of the ALS^C9orf72^ III line was slightly higher at DIV42 than in the other mutant line (*p* = 0.074) indicating that, despite being in agreement with previous findings (Devlin et al., 2015), the drop of firing rate measured upon aging might occur following different dynamics in the two patient lines analyzed in this study.

Simultaneously, the mean interspike interval (ISI) during repetitive firing sequences (defined as mean of all ISIs < 200 ms) also increased over time in ALS^C9orf72^ MN but not in controls (despite remaining comparable between the two genotypes at both time points; Figure 2C). Furthermore, we observed a reduced spike amplitude in ALS^C9orf72^ MN at DIV42 when compared to Healthy controls (Figure 2D).

The HD-MEA technique was chosen since it not only allows the evaluation of the single-electrode parameters mentioned above (resembling alterations on a cellular level), but also of the interconnectivity and network behavior of our MN cultures. Already at DIV21 we could observe a reduction of synchronized neuronal firing in ALS^C9orf72^ MN cultures, which became even more evident in older MN. Indeed, we observed a reduced network burst frequency in young as well as in old ALS^C9orf72^ MN (Figure 2E). In addition, the proportion of spikes fired within network bursts was significantly lower in DIV42 ALS^C9orf72^ MN (Supplementary Figure 1A), whereas the number of spikes inside of each burst at both time points was on average higher than in Healthy MN (Supplementary Figure 1B). Concomitantly, network events at DIV21 lasted significantly longer in mutant MN, whereas the burst duration had significantly dropped at DIV42 to a level comparable to Healthy controls (Supplementary Figure 1C). Fitting to the generally higher firing rate at DIV21 (Figure 2B), also the mean burst peak firing rate of ALS^C9orf72^ MN was increased at the early time point (Supplementary Figure 1D).

Interestingly, we noticed that immature ALS cultures did not show signs of neuronal loss despite being characterized by aberrantly increased firing rate. In fact, we did not find any difference in the levels of leaked LDH (Fujimori et al., 2018) at DIV21 in mutant cultures when compared to Healthy ones. In contrast, LDH levels were significantly higher in ALS^C9orf72^ MN than in controls at DIV42 (Supplementary Figure 2) indicating that, similar to what is observed in humans, signs of neurodegeneration might become detectable at later stages of maturation.

### Time-Dependent Transcriptional Alterations Correlate With the Activity Impairments Observed in ALS^C9orf72^ Cultures

**FIGURE 3:**
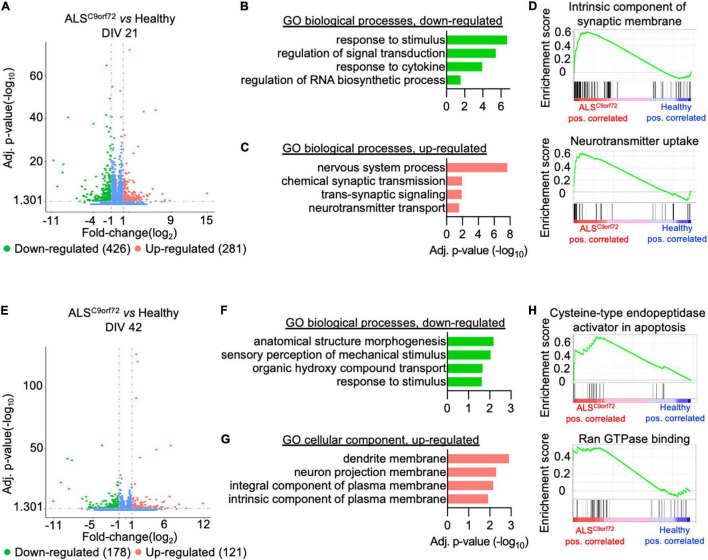
Activity alterations of ALS^C9orf72^ motor neurons are recapitulated by their transcriptional programs. (A) Volcano plot displaying the down- and up-regulated DEGs in ALS^C9orf72^ MN at DIV21. (B) Representative GO biological pathways significantly down- or (C) up-regulated in mutant cultures. (D) GSEA confirming the increased expression of transcripts involved in neuronal firing. (E) Volcano plot displaying the down- and up-regulated DEGs in ALS^C9orf72^ MN at DIV42. (F) Representative GO biological pathways significantly down-regulated in mutant cultures. (G) GO cellular component terms significantly up-regulated in ALS^C9orf72^. (H) GSEA showing increased expression of transcripts involved apoptosis and Ran GTPase binding in aged mutant cultures.

We then analyzed the total transcriptome of Healthy and ALS^C9orf72^ MN at DIV21 and DIV42 to uncover which transcriptional alterations might underlie the activity phenotype observed in ALS^C9orf72^ MN at different stages of maturation. PCA revealed that aging was the variable that contributed the most to the differences observed in the dataset: in fact, PC1 could clearly separate the DIV21 samples from the DIV42 ones (Supplementary Figure 3). When comparing immature mutant MN to Healthy ones, we detected 426 down-regulated and 281 up-regulated differentially expressed genes (DEGs) (Figure 3A and Supplementary Figure 4). Gene ontology (GO) analysis revealed that the down-regulated transcripts clustered in biological processes (BP) linked to stimuli response and, in line with previous findings [reviewed in Zaepfel and Rothstein (2021)], to RNA metabolism (Figure 3B). On the other hand, the up-regulated terms highlighted higher levels of genes involved in synaptic activity and neuronal function (Figure 3C), which were also confirmed in gene set enrichment analysis (GSEA; Figure 3D) and in line with the higher firing rate characterizing the mutant cultures at the same time point in MEA experiments.

Interestingly, when we compared the transcriptome of the two genotypes at DIV42 (Supplementary Figure 5) we found a reduced number of DEGs in ALS^C9orf72^ MN than at the earlier stage (178 down- and 121 up-regulated; Figure 3E), also in agreement with the reduced separation between ALS and Healthy samples observed in the PCA at this time point (Supplementary Figure 3). We found that the enriched down-regulated biological processes were again linked mainly to stimuli response (Figure 3F), while no significant enrichment in BP was found to be linked to the up-regulated genes. Anyway, the GO cellular component showed a significant increase in transcripts involved in neuronal structures (Figure 3G). In addition, GSEA identified increased apoptotic processes in mutant cultures at DIV42, as well as in Ran GTPase binding (Figure 3H), thus confirming increased neuronal vulnerability at this time point.

### Upon Aging, ALS^C9orf72^ Motor Neurons Display Expression Loss of Pre-synaptic Transcripts

We then focused on the transcriptional dynamics occurring upon maturation by comparing the transcriptomes of DIV21 and DIV42 MN within the same genotype. In older cultures from Healthy controls (Supplementary Figure 6A), down-regulated genes significantly enriched in pathways involved in cell differentiation, cell cycle and neuronal development (Supplementary Figure 6B), while we observed a significant up-regulation of terms involved in homeostasis, cell signaling and metabolism when compared to DIV21 MN (Supplementary Figure 6C). Thus, these data confirmed the neuronal maturation occurring over time in Healthy cultures.

**FIGURE 4:**
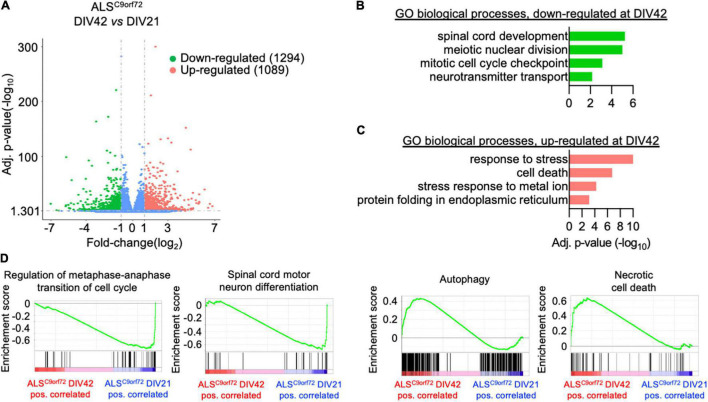
Longitudinal analysis of the ALS^C9orf72^ transcriptome highlights increased neuronal sufferance and reduced synaptic transcripts. (A) Volcano plot displaying the DEGs detected in ALS^C9orf72^ MN upon aging. (B) Representative GO biological pathways significantly down- or (C) up-regulated at DIV42 when compared to DIV21 cultures. (D) GSEA showing enriched correlation of pathways involved in cellular division and spinal cord development with DIV21 MN, while autophagy and cell death are strongly correlated to aged cultures.

In contrast, the changes occurring upon aging in the transcriptomes of ALS^C9orf72^ MN (Figure 4A) highlighted a reduction of terms not only involved in spinal cord differentiation and cell cycle, but also in neurotransmitter transport (Figure 4B), remarkably supporting the loss of neuronal activity observed in MEA experiments over time. In agreement with the increased cellular damage detected by LDH assay at DIV42, we also found increased levels of genes involved in stress and apoptosis (Figure 4C) in older cultures, which were also confirmed by GSEA (Figure 4D). Altogether, our data indicate that mutant cultures are characterized by a time-dependent loss of activity (although reduced synchronous firing was observed already at DIV21), which can be linked to increased stress, apoptosis and reduced expression of synaptic genes occurring upon aging.

**FIGURE 5:**
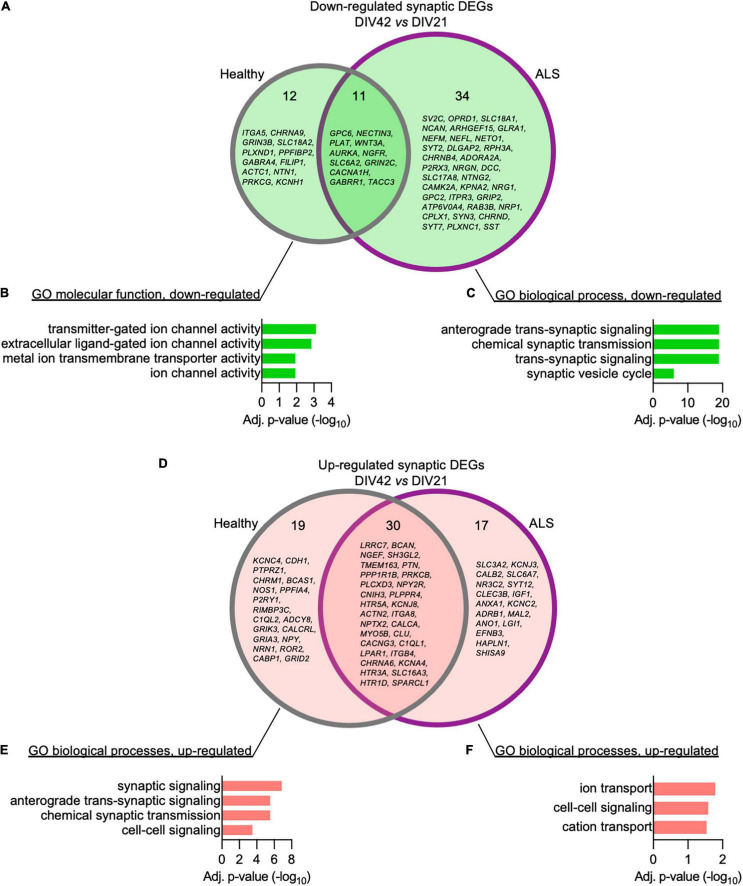
The time-dependent loss of synaptic transcripts in ALS^C9orf72^ motor neurons mainly affects the neurotransmitter vesicle release machinery. (A) Venn diagram showing the genotype-specific synaptic DEGs whose expression decreases over time. (B) Corresponding enrichment analysis performed for Healthy and (C) ALS^C9orf72^ MN. (D) Venn diagram showing the genotype-specific synaptic DEGs whose expression increases over time. (E) Corresponding enrichment analysis performed for Healthy and (F) ALS^C9orf72^ MN.

We then set out to better elucidate how the synaptic transcriptome contributes to the activity-related alterations observed in ALS MN. Using the SynGO analytical tool (Koopmans et al., 2019), we filtered the DEGs whose expression significantly changed over time in Healthy and ALS^C9orf72^ MN and looked at synaptic genotype-specific alterations. At DIV42, we identified 12 down-regulated synaptic DEGs in Healthy and 34 in ALS^C9orf72^ cultures (as well as 11 that were commonly reduced in older neurons of both groups) (Figure 5A). Enrichment analysis revealed that while the down-regulated synaptic DEGs detected in DIV42 Healthy MN were involved in BP linked to ion channel activity (Figure 5B), ALS^C9orf72^ cultures showed a significant reduction in the expression of genes involved in chemical synaptic signaling and synaptic vesicle cycle, such as *RAB3B, SYT2, SYT7, SV2C*, and *SYN3* (Figure 5C), again in remarkable agreement with the MEA data. Interestingly, the enriched terms that were down-regulated in MN from ALS patients appeared to be up-regulated in control cultures (Figures 5D,E), resembling the synaptic maturation occurring over time in healthy controls. In striking parallelism with these results, ALS^C9orf72^ MN were characterized by higher levels of transcripts involved in ion transport, such as the potassium inwardly rectifying channel *KCNJ3* and the voltage-gated potassium channel *KCNC2* (Figure 5F), supporting the role of K^+^ channels in disease manifestation and propagation (Castelli et al., 2021).

### Apamin Rescues the Activity Properties and the Synaptic Transcriptional Program of ALS^C9orf72^ Motor Neurons

**FIGURE 6:**
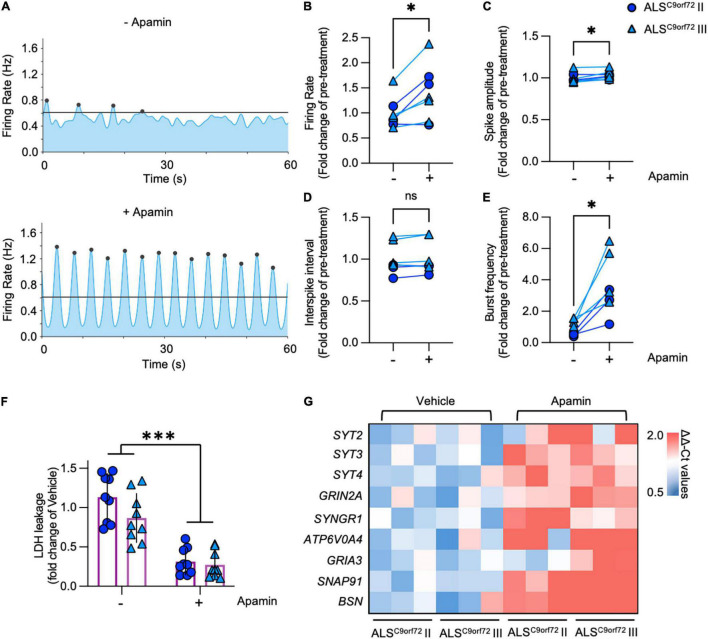
Apamin treatment has a beneficial effect on electrophysiological parameters, cell stress/survival and the expression of synaptic genes in DIV42 ALS^C9orf72^ motor neurons. (A) Representative network activity plots of the same ALS^C9orf72^ MN culture before and after the application of Apamin (lines indicate the network burst detection threshold). (B) Apamin significantly increases firing rate and spike amplitude (C) in DIV42 ALS^C9orf72^ motor neurons, while there was no detectable effect on the interspike interval (ISI) during repetitive firing sequences (D). (E) Apamin-treated ALS^C9orf72^ motor neurons show a higher network burst frequency. (F) Apamin significantly reduces the LDH leakage in ALS^C9orf72^ MN. (G) Heatmap showing the synaptic genes significantly upregulated upon Apamin treatment. *p < 0.05; ^***^p < 0.001.

Our transcriptome analysis suggested that pre-synaptic alterations affecting the neurotransmitter release might contribute to the activity reduction and impaired network burst firing observed in mutant neurons. Considering that K^+^ channel blockade increases neuronal activity, positively modulates the generation of synchronized bursts (Mahrous and Elbasiouny, 2017) and exerts a neuroprotective effect in human ALS MN (Naujock et al., 2016; Castelli et al., 2021; Catanese et al., 2021), we evaluated the impact of the SK channel blocker Apamin on the electrophysiological properties of DIV42 ALS^C9orf72^ MN. In line with the role of the SK channels in increasing the firing gain by controlling the after-action potential hyperpolarizing current (AHP; Manuel et al., 2006), Apamin treatment significantly increased the firing rate of mutant neurons (Figures 6A,B). In addition, we found a minor albeit significant increase in the spike amplitude of treated neurons (Figure 6C), whereas no significant alteration was observed in the interspike interval during repetitive firing sequences between both experimental conditions (ISI, Figure 6D).

In addition to the parameters detectable on the single electrode level, we also evaluated the effects of K^+^ channel blockade on the network properties of ALS^C9orf72^ cultures. Most importantly, Apamin significantly increased the burst frequency of mutant MN (Figure 6E), which was earlier found to be reduced in comparison to Healthy controls (Figure 2E). Furthermore, the percentage of spikes within bursts as well as the burst peak firing rate were significantly increased (Supplementary Figures 7A,B), whereas burst duration was lower in the post-treatment condition (Supplementary Figure 7C) and no significant effect was observed in the number of spikes per burst (Supplementary Figure 7D).

Notably, increasing neuronal activity in hyperactive DIV21 ALS^C9orf72^ cultures with Apamin did not exert any effect on the MN survival (Supplementary Figure 8), while treating DIV42 mutant cells with the K^+^ channel blocker significantly reduced the levels of leaked LDH (Figure 6F), in agreement with the neuroprotective role of Apamin in ALS^C9orf72^ MN (Castelli et al., 2021; Catanese et al., 2021).

We then investigated the effect of increased neuronal firing on the synaptic transcriptome of ALS^C9orf72^ MN (Figure 6G): we found that Apamin rescued the levels of *SYT2* and *ATP6V0A4*, whose expression was reduced in aging mutant cultures. In addition, treated neurons displayed higher levels of the *SYT3* and *SYT4* genes, which encode for proteins involved in neurotransmitter release, as well as of the *GRIN2A, SYNGR1, GRIA3, SNAP91* and *BSN* transcripts, which belong to the LcMN cluster (Figure 1B). This indicates that Apamin exerts a neuroprotective effect by re-establishing burst firing properties in ALS-related MN and driving them toward a more synaptically mature and functional status.

## Discussion

The dispute concerning the hyper- and hypoexcitability theories has been dividing the ALS research community for the last decade, as contrasting evidence obtained with different experimental models has been published in support of both lines of thought. Kuo and colleagues showed indeed that MN prepared from embryos of the SOD1(G93A) mouse model are hyperexcitable (Kuo et al., 2004), while an independent study linked reduced dendritic complexity to the increased activity observed in SOD1(G93A) primary MN when compared to WT cells (Martin et al., 2013). In contrast, electrophysiological recordings performed *in vivo* in the same mouse model, as well as in FUS(P525) mice (Sharma et al., 2016), showed that fast fatigable (FF) MN, the most vulnerable subpopulation of MN in ALS (Delestrée et al., 2014), are hypoexcitable already at a pre-symptomatic stage of disease progression (Martínez-Silva et al., 2018). Thus, reduced excitability might represent a specific early pathological feature characterizing the adult vulnerable cells. For the sake of clarity, it is also to be mentioned that opposing results were obtained depending on the experimental settings used for the electrophysiological investigations *in vivo* (Martínez-Silva et al., 2018; Ba̧czyk et al., 2020; Jensen et al., 2020), making it difficult to interpret the evidence focusing on activity abnormalities in ALS.

In this puzzling scenario, the investigation of activity-related alterations occurring in ALS using human MN from iPSC has further produced contradictory results, mostly arising from the maturation state considered in the different studies. Hyperactivity has been in fact mainly observed in ALS-related cultures at the early stages of differentiation, while reduced excitability seems to be characteristic of more mature mutant MN (Sareen et al., 2013; Wainger et al., 2014; Devlin et al., 2015). Our results are in remarkable agreement with this theory, as we detected significantly higher activity in immature C9orf72-mutant cultures and observed a time-dependent loss of firing properties upon aging. In contrast to what was observed by Wainger and colleagues, but in agreement with previous findings reporting neuronal loss in mutant culture only after the fifth week *in vitro* (Naujock et al., 2016; Fujimori et al., 2018; Catanese et al., 2021), the aberrant firing characterizing DIV21 ALS^C9orf72^ MN did not appear to affect their survival rate. This suggests that either mutant MN are more resistant to disease at this immature state, or that at this early time point of *in vitro* differentiation a pool of proliferating neural precursor cells might still be present and compensate for the ongoing loss of already differentiated neurons. Our data are more in favor of the latter hypothesis: the transcriptional changes from DIV21 to DIV42 clearly highlighted a reduced cell proliferation in aged cultures and, after 3 weeks of differentiation using the same protocol of this study, Olig2-positive MN precursors represent almost the 20% of the cells in culture (Catanese et al., 2021). Nevertheless, we cannot completely exclude that mutant MN might be indeed more resistant to ALS-related phenotypes when immature. First, DIV21 hyperactive mutant MN did not show signs of cell death despite the accumulation of aberrant aggresomes already at this stage, suggesting an underlying higher level of cellular stress (Catanese et al., 2019; Catanese et al., 2021). In fact, hyperactivity has been also previously linked to accumulation of cytotoxic aggregates (Weskamp et al., 2020). Second, further increasing neuronal firing with Apamin at DIV21 did not trigger neuronal loss. In both cases, the presence of immature and still differentiating cells might strongly contribute to the observed hyperactivity characterizing early mutant cultures, making these results of questionable relevance as their maturity does not match the one of motor neurons affected in living patients. In fact, hyperactivity seems to be a specific feature of S-type motor neurons, which are more resistant to ALS progression (Leroy et al., 2014). Moreover, it is still unknown whether hiPSC-derived cultures might represent the different degree of vulnerability to neurodegeneration by differentiating into distinct MN subtypes (Saxena et al., 2013; Delestrée et al., 2014). This possibility, although intriguing, is rather unlikely since iPSC-derived neurons resemble a general embryonic state (Ho et al., 2016) and a specific protocol to obtain the different subpopulations has still not been described. Nevertheless, a patient-specific approach such as personalized iPSC modeling could help to identify the precise timing of phenotype manifestation and confer even more translational relevance to the results obtained *in vitro.* Indeed, the reduction of firing rate occurred at different pace in the lines we analyzed, highlighting differences that might be addressed by such a patient-specific strategy.

But where does the early hyperactivity originate from? Mitochondrial inhibition induces the opening of ATP-sensitive potassium channels, triggering a transient membrane hyperpolarization that is followed by a non-reversible depolarization (Riepe et al., 1992). Since mitochondrial dysfunction has been also observed in young C9orf72-mutant MN (Mehta et al., 2021) and given the low synaptic density observed in immature MN, it is reasonable to speculate that the increased activity of ALS^C9orf72^ MN might be related to their intrinsic excitability (Leroy et al., 2014; Wainger et al., 2014) rather than to proper synaptic activity. In fact, despite displaying significantly higher rates of spontaneous firing than controls, DIV21 ALS^C9orf72^ cultures showed less synchronized network activity already at this early time point, resembling similar evidence obtained with human C9orf72-mutant cortical neurons (Perkins et al., 2021). Thus, the synaptic microenvironment might represent a crucial hub for the development and progression of disease-related phenotypes. Based on these considerations, we aimed at assessing the pattern of MN maturation from the synaptic perspective. Since synapses are indeed a critical structure involved in neuronal activity, we reasoned that identifying a “synaptic signature” defining a mature motor neuron might reveal helpful information for assessing activity-related phenotypes in ALS using hiPSC. Indeed, synaptic remodeling is a critical step during neuronal maturation (Sakai, 2020). In addition, despite the intense research performed using this particular model, a commonly accepted definition of a mature and physiologically relevant status for iPSC-derived MN, based on biochemical and physiological evidence, is still missing. Our approach, based on transcriptome analysis at the different steps of MN differentiation and maturation, revealed for the first time a cluster of synaptic genes defining the state of adult and mature motor neurons. This dataset represents not only a valuable tool to be applied by other researchers in the field of ALS, but it might also contribute to a better understanding of the spinal cord synaptic network and be applied to other diseases affecting MN, such as Spinal Muscular Atrophy (SMA), or even other areas of the central nervous system. In fact, this cluster contained several genes such as *BSN, DNAJC6, GRIA4, SNAP25*, and *SYNJ1* that have been linked, causally or as risk factor, to different neurological conditions when mutated (Quadri et al., 2013; Rohena et al., 2013; Olgiati et al., 2016; Martin et al., 2017; Yabe et al., 2018). Moreover, altered excitability and synaptic composition represents a pathological feature commonly shared by several neurodegenerative diseases such as Alzheimer’s, Huntington’s and SMA [reviewed by Bae and Kim (2017)], suggesting that the hereby presented maturity-related synaptic signature might represent an interesting methodological entry point for the investigation of a broader spectrum of conditions.

Here, by assessing the neuronal activity of ALS cultures according to their synaptic maturity, we noticed that the spontaneous firing of mutant MN decreased and that the ability of firing synchronized bursts worsened even further upon aging. Importantly, the more vulnerable FF MN tend to fire high-frequency bursts (Burke, 1980) and, confirming the relevance of our findings on this physiological property, impaired bursting has indeed been detected by performing *in vivo* MN recordings in an ALS mouse model (Hadzipasic et al., 2016).

Together with the altered ability of firing synchronized bursts, suggesting synaptic imbalance (Leleo and Segev, 2021), the early hyperactivity detected in ALS^C9orf72^ cultures was also matched by increased expression of synaptic transcripts and in line with the up-regulation of GluR1 at DIV17 detected by Shi and colleagues in C9orf72-mutant iMN (directly converted from fibroblasts; Shi et al., 2019). Though this early upregulation might appear in discordance with previously described reduced levels of synaptic genes in ALS MN (Sareen et al., 2013; Hall et al., 2017; Catanese et al., 2021; Mehta et al., 2021), this seems to be related to the maturation state investigated. In addition, it has to be considered that the different strategies used to obtain MN from hiPSC might still represent a strong source of biases as they can lead to differences in culture composition, neuronal yield and even delayed neuronal maturation (Muratore et al., 2014; Schenke et al., 2020). This again lies in favor of our synaptic “maturity signature,” which might be used to identify the most suitable time points to investigate ALS-linked phenotypes and compare them to datasets generated by using different protocols. Indeed, several previous studies analyzing ALS transcriptomes have been performed at stages of maturation where MN already showed clear signs of suffering and death (Kiskinis et al., 2014; Fujimori et al., 2018), leaving unclarified whether the transcriptional alterations observed represent the cause or direct consequences of the ongoing degeneration. Our strategy, involving transcriptional analysis based on the synaptic maturity of cultured MN, emphasized the importance of performing longitudinal studies to highlight relevant alterations in ALS (Ho et al., 2021). We indeed show a time-dependent loss of synaptic gene expression as neurons mature, accompanied by increased expression of transcripts involved in autophagy and neuronal death. Thus, our transcriptomic data indicated that altered expression of synaptic transcripts actively contributes to the disease progression observed *in vitro*, in line with the idea that vulnerable MN lose synaptic contacts and firing properties already at the pre-symptomatic stage (Ba̧czyk et al., 2020).

Of note, the increased cell death observed at the later time point of investigation might also contribute to the reduction of firing properties detected in our longitudinal MEA recordings. Nevertheless, we believe that the neuronal loss ongoing in mutant cultures can only partially explain their loss of activity. If a massive loss of MN would occur in mutant cultures, a general reduction in both pre- and postsynaptic markers (including genes encoding for scaffold proteins) would be expected. In contrast, our results are in agreement with previous works describing a loss of synaptic transcripts (Mehta et al., 2021) even before neuronal death (Catanese et al., 2021), and strengthen the idea of pathological alterations affecting the synaptic structures being a shared feature of different ALS models (Hall et al., 2017; Ba̧czyk et al., 2020; Jensen et al., 2020; Catanese et al., 2021). In this context, restoration of synaptic composition through enhanced neuronal activity proved neuroprotective not only in ALS (Saxena et al., 2013; Ba̧czyk et al., 2020; Catanese et al., 2021), but also in SMA (Simon et al., 2021). Mechanistically, it appears that the transcriptional feedback loop set in motion by sustained neuronal firing and increased bursting might activate neuroprotective transcription factors, such as CREB (Lee et al., 2005; Catanese et al., 2021), thereby increasing the transcription of synaptic genes and contributing to the preservation of synaptic contacts. This actually appears to be independent from the method used to increase neuronal firing since chemogenetic, optogenetic, as well as pharmacological strategies have improved the disease progression of both *in vivo* and *in vitro* ALS models (Saxena et al., 2013; Naujock et al., 2016; Ba̧czyk et al., 2020; Catanese et al., 2021). Notably, in our previous work we showed that the neuroprotective effect of increased activity was lost when exceeding the ideal concentration of K^+^ channel blockers or at higher frequency of optogenetic stimulation (Catanese et al., 2021). Thus, neuroprotection through enhanced MN firing might be achieved in a dose-dependent manner.

## Conclusion

In conclusion, our study provides *proof of principle* data not only indicating a fundamental role played by the synaptic transcriptome in defining MN maturity, but also reinforcing the idea that synaptic integrity represents a *conditio sine qua non* to maintain proper neuronal activity and counteract neurodegenerative processes. K^+^ channel blockers are a class of compounds that proved beneficial in different motor neuron diseases (Naujock et al., 2016; Catanese et al., 2021; Simon et al., 2021) by increasing burst firing properties of MN (Mahrous and Elbasiouny, 2017) and activating different cellular mechanisms converging on synaptic strengthening in murine and human models. In particular, it has been shown that FF MN fire rarely, in bursts and express high-levels of SK channels. Thus, interventions aimed at preserving a physiological synaptic structure and functionality, as well as restoring proper firing properties, might represent an effective strategy to delay, if not prevent, motor neuron degeneration (Roselli and Caroni, 2015).

All in all, despite being based on a restricted number of hiPSC lines, our work represents an entry point for the development of strategies aimed at re-establishing neuronal physiology and synaptic composition as a treatment for ALS. This approach should be further tested by expanding the analysis and the application of the aging-related synaptic signature to a broader and more heterogeneous cohort of ALS cases. In fact, despite being the most frequent genetic cause of motor neuron disease, C9orf72 mutations do not explain the whole spectrum of pathological phenotypes observable in ALS. Moreover, the exact effect of the genetic defects on the observed alterations has still not been dissected from the broad differences highlighted by the comparison to healthy controls. Thus, challenging ALS-related MN with mutations in different genes, together with using CRISPR-Cas9 technology to create isogenic controls, might strongly broaden the relevance of our results. Since synaptic defects have been indeed described in the presence of different pathogenic mutations (Hall et al., 2017; Catanese et al., 2021), this strategy might even contribute to the identification of pathological features and therapeutical targets shared across the heterogenous landscape of ALS.

## Data Availability Statement

The original data concerning the RNAseq experiments in hiPSC-derived MN presented in this study are publicly available at Gene Expression Omnibus (GEO) repository under the accession number GSE201407.

## Ethics Statement

All procedures with human material have been performed within the context of the German Network for Motor Neuron Diseases (MND-NET), which has been approved by the Ethical Committee of Ulm University (approval Nr. 19/12). Experiments have been performed in compliance with the guidelines of the Federal Government of Germany and after receiving informed consent for the study from all participants. The use of human material was approved by the Declaration of Helsinki concerning Ethical Principles for Medical Research Involving Human Subjects, and experiments were performed according to the principles set out in the Department of Health and Human Services Belmont Report.

## Author Contributions

AC conceived the project. DS, RH, TB, and AC planned the experiments. DS, SR, MS, AA, and AC performed the experiments. DS, RH, and AC analyzed the data. DS, SR, and AC wrote the manuscript. AL, RH, and TB revised the manuscript. TB and AC provided the fundings. All authors contributed to the article and approved the submitted version.

## Conflict of Interest

The authors declare that the research was conducted in the absence of any commercial or financial relationships that could be construed as a potential conflict of interest.

## Publisher’s Note

All claims expressed in this article are solely those of the authors and do not necessarily represent those of their affiliated organizations, or those of the publisher, the editors and the reviewers. Any product that may be evaluated in this article, or claim that may be made by its manufacturer, is not guaranteed or endorsed by the publisher.

## Supplementary Material

Supplementary Table 1 Detailed statistical analysis in longitudinal MEA experiments.

Supplementary Table 2 Ranking of the synaptic genes according to their loading scores in PC1.
