# Supplementary figures and images for: Aging-Dependent Altered Transcriptional Programs Underlie Activity Impairments in Human C9orf72-Mutant Motor Neurons
Source: Front Mol Neurosci. 2022 Jun 14;15:894230. doi: 10.3389/fnmol.2022.894230 (PMC9237792; doi:10.3389/fnmol.2022.894230)

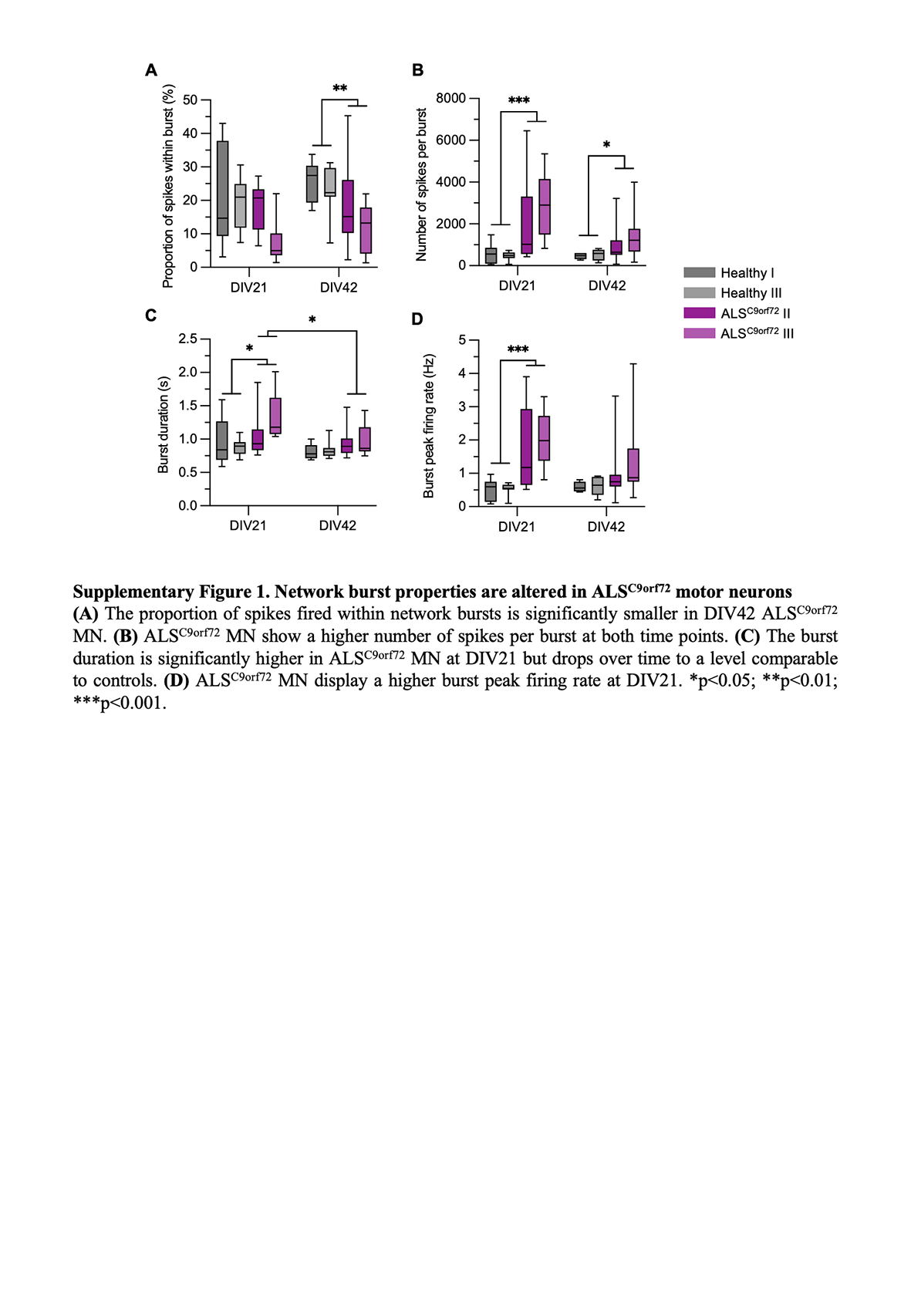

Supplement: Supplementary file 1 [file Image_1.tiff]

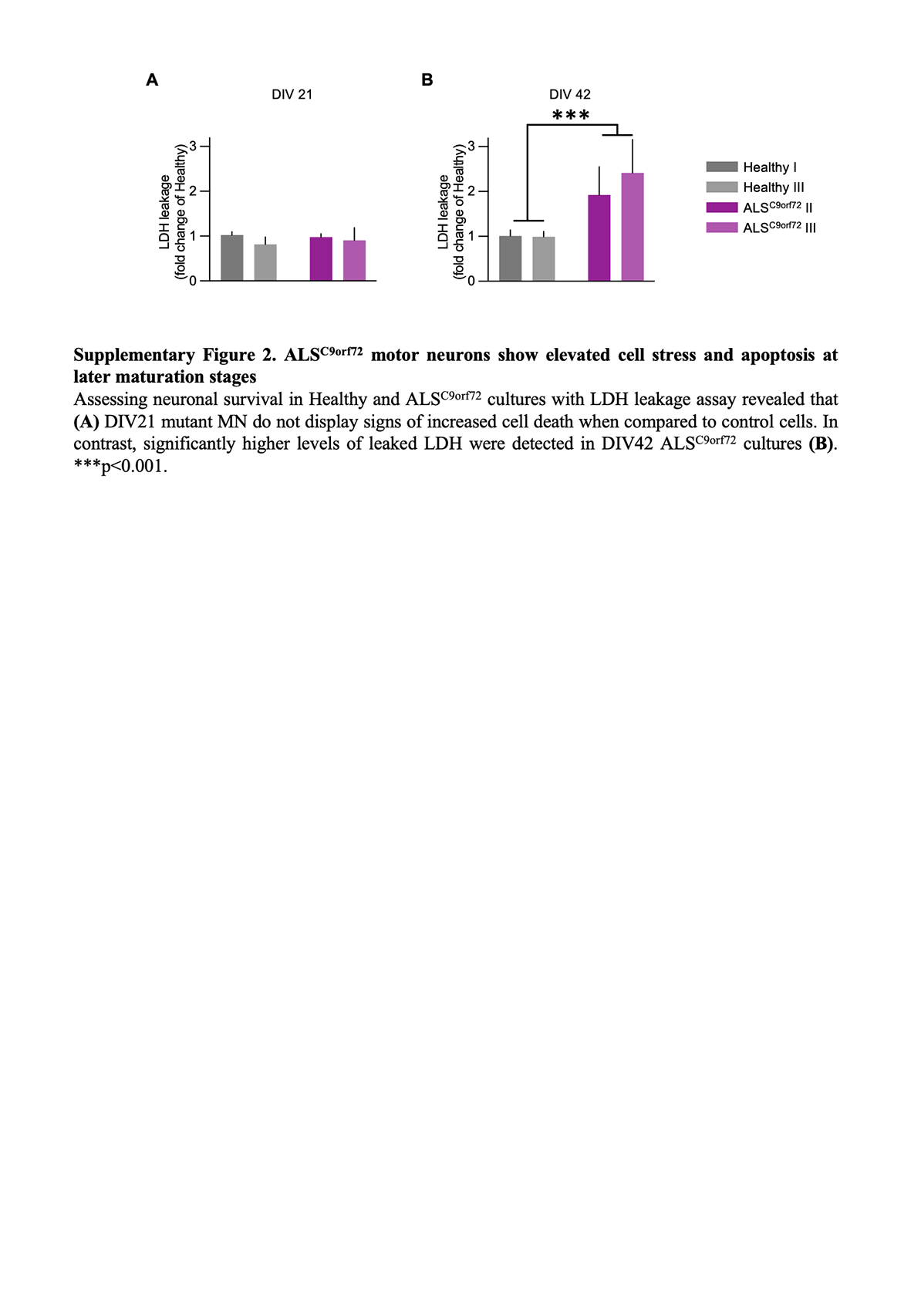

Supplement: Supplementary file 2 [file Image_2.tiff]

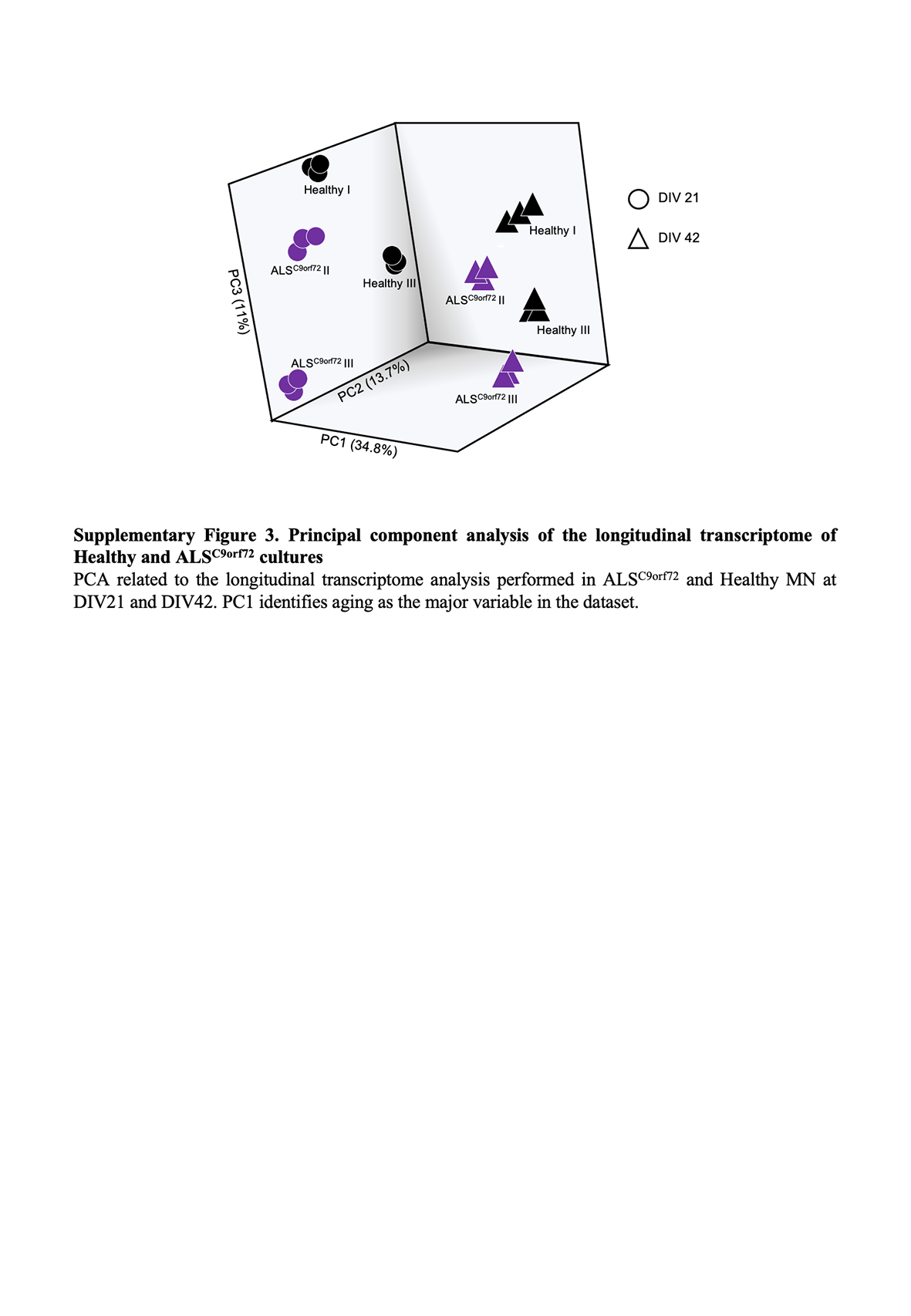

Supplement: Supplementary file 3 [file Image_3.tiff]

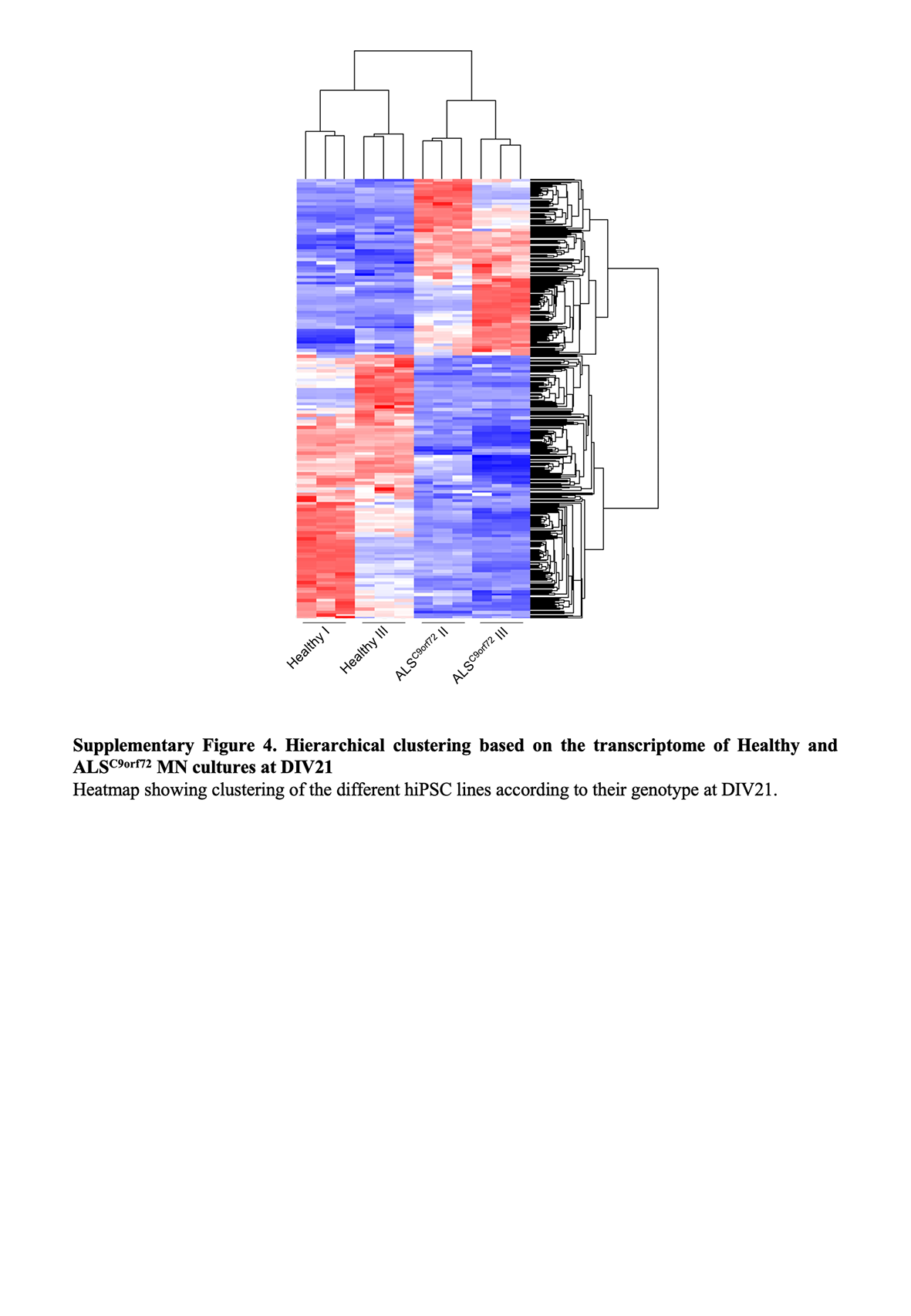

Supplement: Supplementary file 4 [file Image_4.tiff]

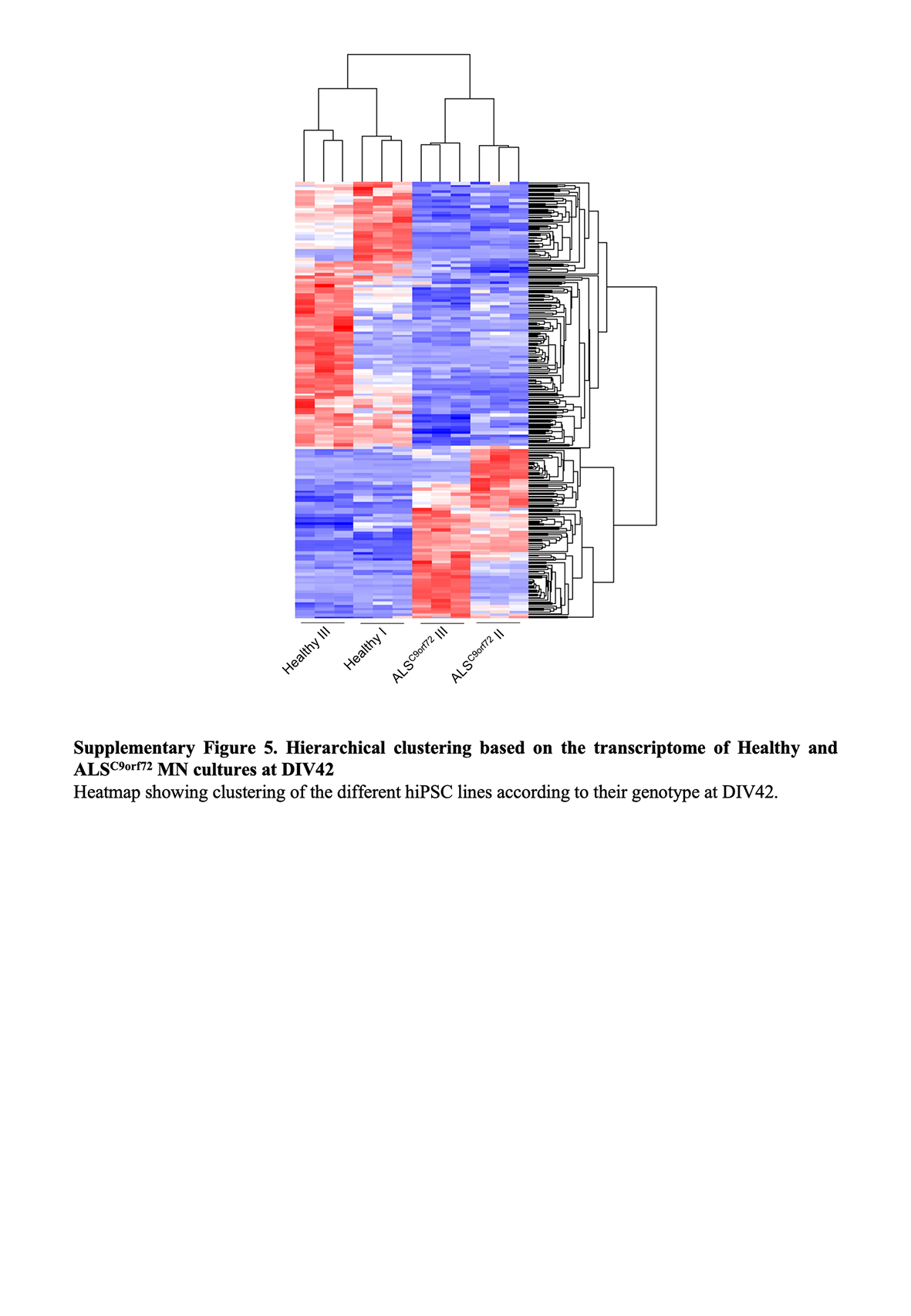

Supplement: Supplementary file 5 [file Image_5.tiff]

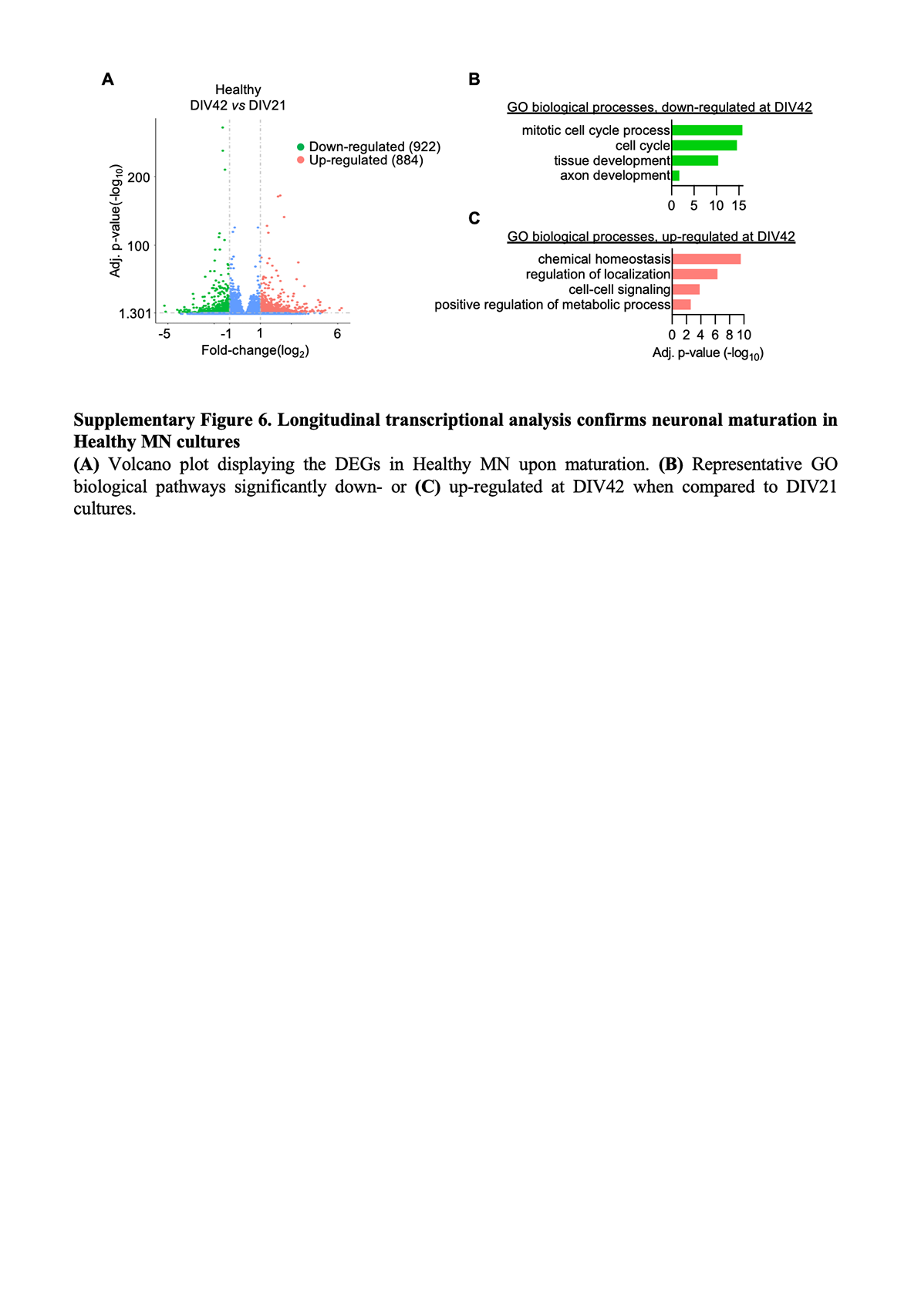

Supplement: Supplementary file 6 [file Image_6.tiff]

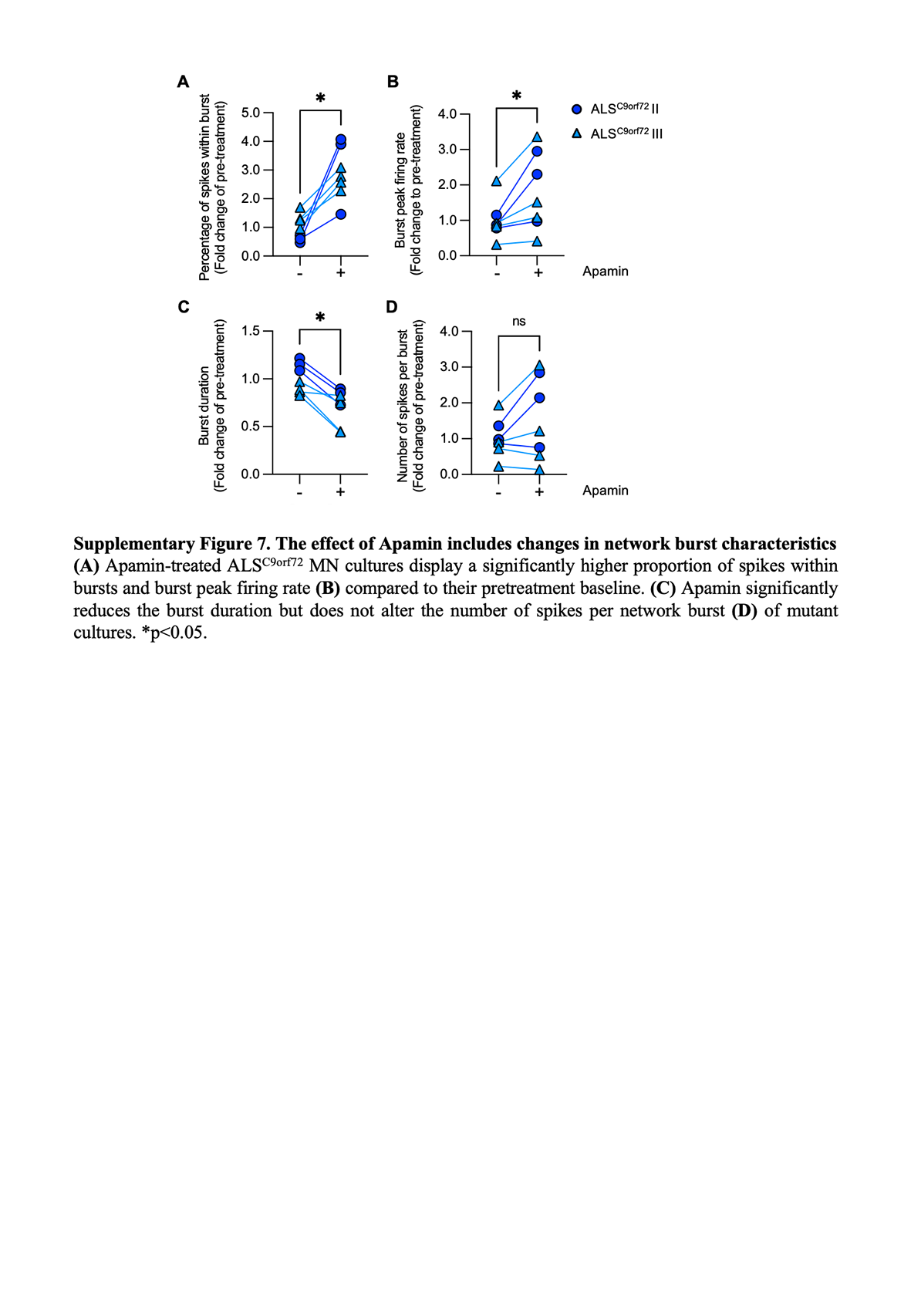

Supplement: Supplementary file 7 [file Image_7.tiff]

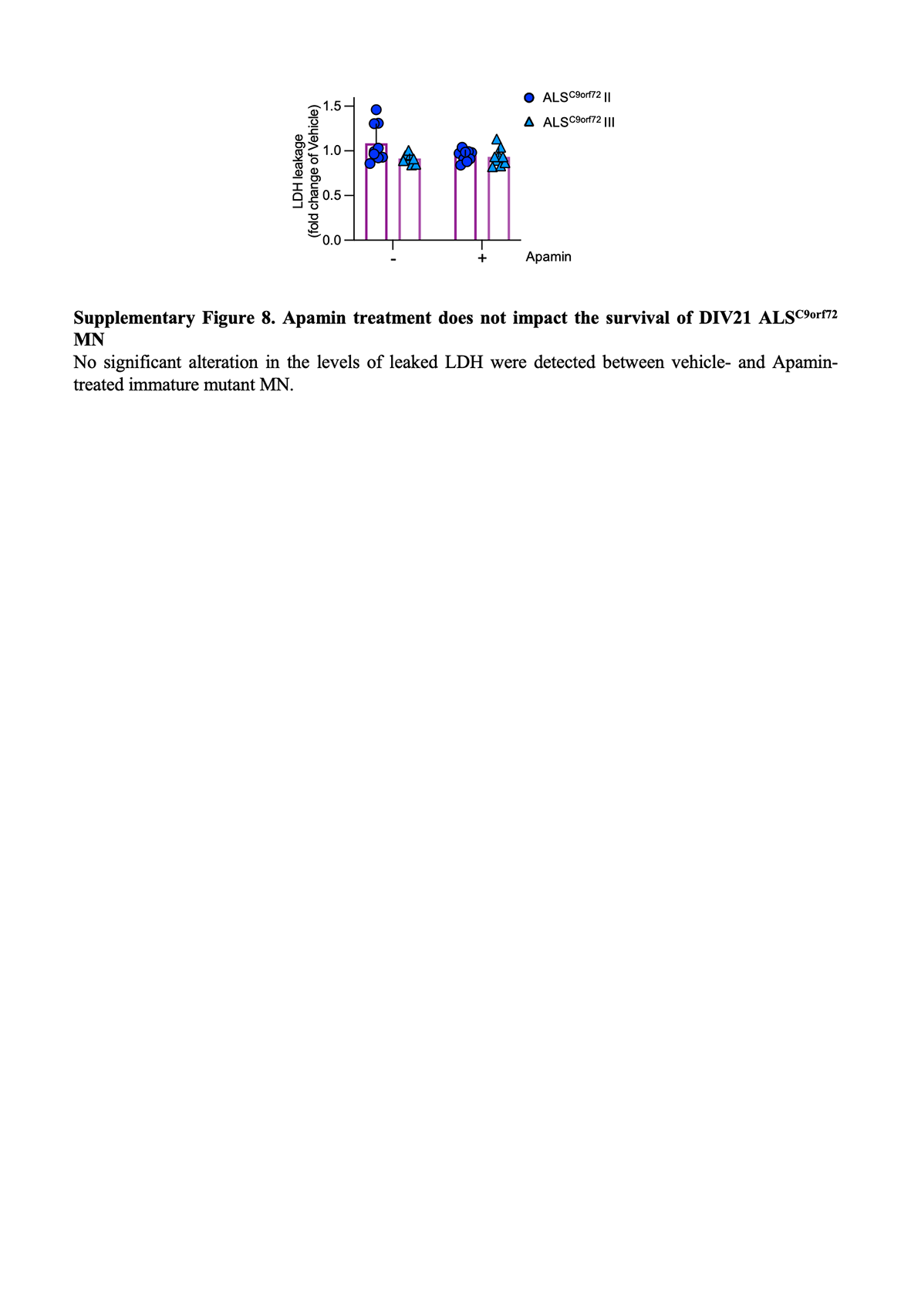

Supplement: Supplementary file 8 [file Image_8.tiff]
